# The catastrophic association of prosthetic mitral valve thrombosis and hemorrhagic stroke

**DOI:** 10.21542/gcsp.2023.31

**Published:** 2023-09-30

**Authors:** Humberto Morais, Mauer A.A. Gonçalves

**Affiliations:** 1 Centro de Estudos Avançados em Educação e Formação Médica, Faculdade de Medicina da Universidade Agostinho Neto, Luanda, Angola; 2 Departamento de Cardiologia. Hospital Militar Principal/Instituto Superior, Luanda, Angola; 3 Luanda Medical Center, Luanda, Angola

## Abstract

Prosthetic valve thrombosis is a serious complication of valve replacement associated with a high mortality rate. Stroke may be the first symptom of prosthetic valve thrombosis. We present the case of a patient who visited the emergency department with symptoms of dysarthria and left hemiparesis. An examination revealed an ischemic stroke with hemorrhagic transformation, stemming from a thrombosis of their mitral valve prosthesis, which progressed to the patient’s death. We emphasize the difficulty in the therapeutic and diagnostic management of these patients.

## Introduction

Twenty percent of patients with prosthetic heart valves have a stroke, despite anticoagulant treatment^1^. Furthermore, stroke may be the first form of clinical presentation of prosthetic valve thrombosis (PVT)^2^. The main pathogenic factors for PVT were identified, including the type of prosthesis, prosthesis in the mitral position, presence of atrial fibrillation, atrial dilation, ventricular dysfunction, multivalvular replacements, and pregnancy. However, the most common cause is a subtherapeutic level of anticoagulation^3^.

The therapeutic strategy for PVT is influenced by the prosthesis location, the valve obstruction severity, and the patient’s clinical status. In addition, the ACC/AHA and ESC guidelines are divergent: the ACC/AHA guidelines consider surgery or the use of fibrinolytic therapy (slow infusion of 25 mg tissue plasminogen activator for 6 to 24 h without bolus) as a comparable initial approach (Class I). ESC guidelines, on the other hand, prefer surgery and recommend fibrinolytic therapy (Class IIa) when surgical risk is considered high/unavailable or for right valve thrombosis, using a standard dose of recombinant tissue plasminogen activator (10 mg bolus followed by 90 mg over 90 min with unfractionated heparin)^4^.

The objective of the present report is to describe a case of a patient who went to the emergency department due to dysarthria and left hemiparesis, in which an ischemic stroke with hemorrhagic transformation was found, as the initial presentation of thrombosis of the mitral valve prosthesis. We also emphasize the difficulty in the therapeutic and diagnostic management of these patients.

## Clinical case

A 47-year-old male patient with a history of medicated and controlled arterial hypertension; valve replacement with the placement of a mechanical prosthesis in the mitral position three years before, and was undergoing oral anticoagulation with warfarin.

According to his wife’s information, the patient was fine up to 12 h before coming to the emergency room when he started with speech disorders, deviation of the labial commissure to the left, and decreased muscle strength in the left hemibody.

On physical examination, the patient was conscious, oriented, and aphasic. Pulse: 69 bpm. BP: 133/97 mmHg FR: 22 cpm. Cardiac auscultation without prosthesis noise, without murmurs. Pulmonary auscultation maintained vesicular murmur, no adventitious sounds Abdomen: globose at the expense of the adipose panicle without palpable visceromegaly. Lower limbs: symmetrical, without edema. Summary neurological examination the patient was calm, conscious, oriented, aphasic, decreased muscle strength of the left hemibody, without meningeal signs.

**Figure 1.**
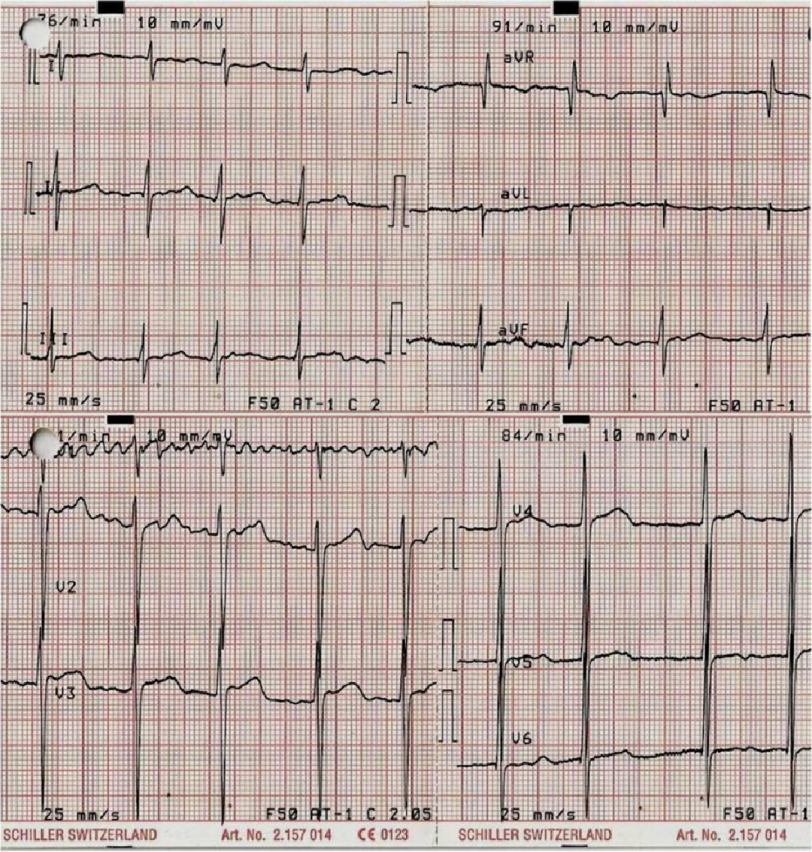
12-lead electrocardiogram reveals atrial fibrillation with controlled ventricular response and signs of left ventricular hypertrophy.

Analytical control at admission showed leukocytosis with neutrophilia, a prothrombin time of 20 s with a subtherapeutic INR of 1.8, and cholesterol of 236 mg/dl without other changes. The ECG revealed atrial fibrillation with controlled ventricular response and signs of left ventricular hypertrophy (Figure 1).

**Figure 2.**
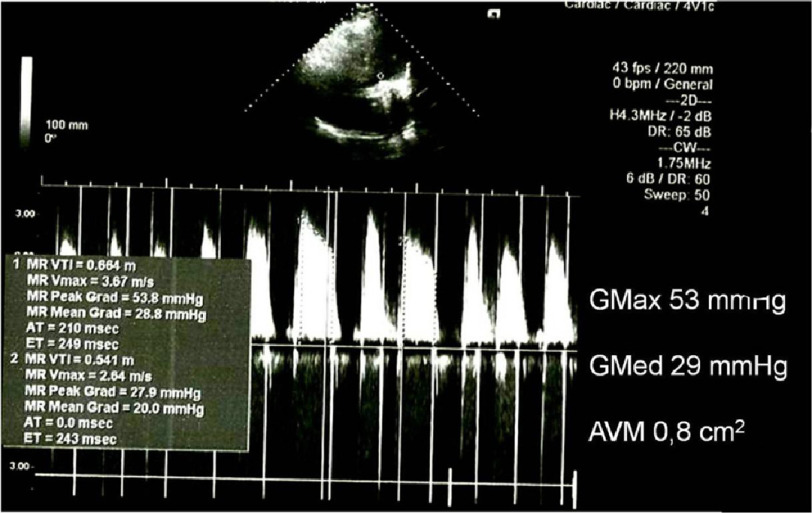
Doppler study of the mitral valve prosthesis revealed a maximum and mean transvalvular gradient of 53 and 29 mmHg, respectively.

The transthoracic echocardiogram, performed 72 h after admission, revealed a poor acoustic window, dilated left atrium (6.25 cm), and normal-sized left ventricle with decreased left ventricular ejection fraction (LVEF) (LVEF 28%). The Doppler study of the mitral valve prosthesis revealed a maximum and mean transvalvular gradient of 53 and 29 mmHg, respectively, suggesting severe obstruction of the valve prosthesis (Figure 2).

**Figure 3.**
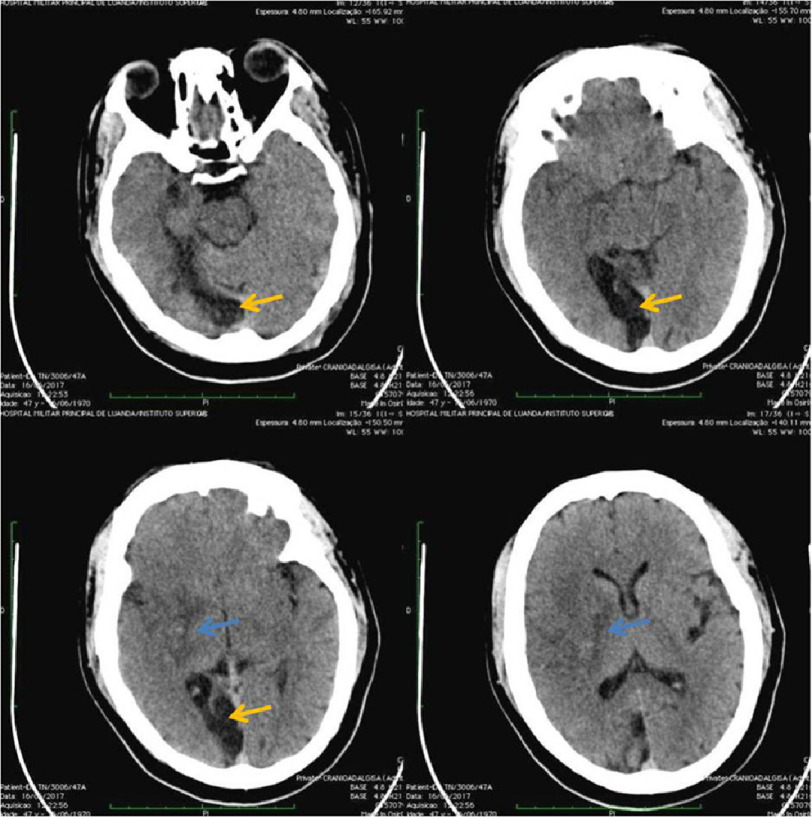
Cranioencephalic computed tomography reveals intraparenchymal hematoma undergoing resorption in the right nucleus capsular topography with surrounding edema (blue arrow); two encephalomalacia foci in the right cerebral hemisphere (yellow arrow); the findings suggest subacute stroke, coexisting foci of chronic stroke.

Cranioencephalic computed tomography revealed intraparenchymal hematoma undergoing resorption in the right nucleus capsular topography with surrounding edema; two encephalomalacia foci in the right cerebral hemisphere; the findings suggest a subacute stroke, with coexisting foci of chronic stroke (Figure 3).

**Figure 4.**
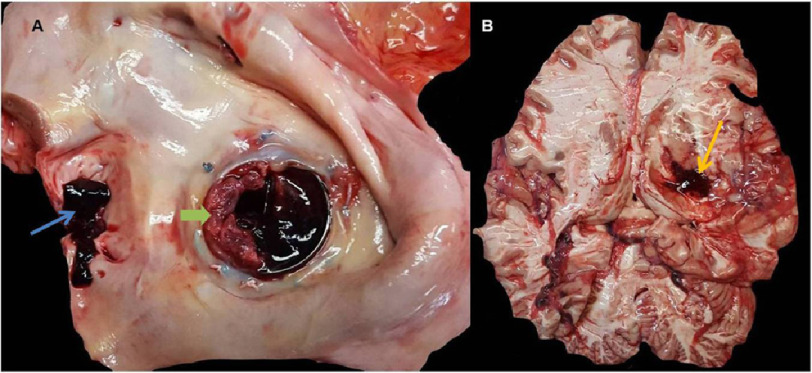
Gross specimen reveals A- prosthesis thrombosis (green arrow) and thrombus in the left atrial appendage (blue arrow). B - Intracranial hemorrhage (yellow arrow).

The patient was medicated with enalapril 20 mg 1 tablet 12/12 h, amlodipine 10 mg 1 tablet/day, mannitol 20% 100 ml 4/4 h, furosemide 20 mg after mannitol. A control cranioencephalic CT performed 48 h after admission showed the presence of extensive cerebral edema. On the third day of hospitalization, the patient underwent irreversible cardiorespiratory arrest. The autopsy confirmed the diagnosis of obstruction of the mitral valve prosthesis and ischemic stroke with hemorrhagic transformation (Figure 4).

## Discussion

Prosthetic valve thrombosis (PVT) is a serious complication of valve replacement and is associated with a high mortality rate. The clinical presentation of PVT can be variable. Patients with TPV may present with symptoms such as dyspnoea, decreased exercise capacity, palpitations, chest pain, vertigo, or stroke - as in the case presented here^5^.

When there is initial suspicion of PVT, a careful physical examination should be performed, with special attention to the muffling or disappearance of prosthesis noises and the appearance of a new regurgitant or obstructive murmur. Initial diagnostic evaluation includes transthoracic echocardiography and cine-fluoroscopy for mechanical valves^6^.

Transthoracic Doppler echocardiography is the most commonly used imaging technique. PVT may be suspected with increased transvalvular gradients. However, this measurement is non-specific as the gradient can be increased by other conditions. On the other hand, normal gradients do not rule out an obstruction in the presence of left ventricular dysfunction or hypovolemia^7^.

Cinefluoroscopy is an important part of the diagnostic evaluation of a suspected PVT, but this technique is not useful for identifying non-obstructive PVT or differentiating pannus from thrombus^6,7^. Transesophageal echocardiography (TTE) is the most sensitive diagnostic tool for identifying an abnormal cardiac mass. Furthermore, TTE provides important additional information to guide therapy and is often performed to complete the investigation^6^. More recently, the diagnostic use of real-time three-dimensional TEE proved to be useful and comprehensive in the assessment of PVT (number, size, and precise location)^8^.

The therapeutic strategy is influenced by the prosthesis’s location, the valve obstruction’s severity, and the patient’s clinical status. The ACC/AHA and SEC Guidelines differ on the initial therapeutic option of surgery *vs* fibrinolytic therapy^4^. In the case presented here, the use of thrombolytics was contraindicated due to the presence of intracranial hemorrhage. Surgical treatment in the acute phase of a stroke may be considered a viable option, particularly in young people with ischemic stroke^9^. In our case, the echocardiogram was performed 72 h after admission, and the patient died a few hours later.

## What have we learned?

• In patients with valve prostheses who present to the emergency department with a stroke, an echocardiogram must be performed within the first few hours to exclude prosthetic valve thrombosis.
• The concurrent existence of hemorrhagic stroke and prosthetic valve thrombosis poses serious difficulties to the care team in terms of treatment.

## AUTHOR STATEMENT

Study design: HM and MAAG

Data collection: HM

Writing of the manuscript: HM and MAAG

Revisions and approval of the final manuscript: All authors

## Conflicts of Interest

The authors declare no conflicts of interest and no specific funding sources for this work.

## Consent

A written voluntary informed consent was obtained from the patient’s family to publish the case in the journal. The patient’s identity, contact details, and address will not be disclosed.
